# Tissue Pathogens and Cancers: A Review of Commonly Seen Manifestations in Histo- and Cytopathology

**DOI:** 10.3390/pathogens10111410

**Published:** 2021-10-30

**Authors:** Tzy Harn Chua, Lavisha S Punjabi, Li Yan Khor

**Affiliations:** 1 Department of Anatomical Pathology, Singapore General Hospital, Singapore 169856, Singapore; tzyharn.chua@mohh.com.sg (T.H.C.); lavisha.punjabi@mohh.com.sg (L.S.P.); 2 Duke-NUS Medical School, Singapore 169856, Singapore

**Keywords:** pathogen, cancer, virus, bacteria, parasites, pathology, cytology

## Abstract

Tissue pathogens are commonly encountered in histopathology and cytology practice, where they can present as either benign mimickers of malignancy or true malignancies. The aim of this review is to provide a timely synthesis of our understanding of these tissue pathogens, with an emphasis on pertinent diagnostic conundrums associated with the benign mimickers of malignancy that can be seen with viral infections and those which manifest as granulomas. The oncogenic pathogens, including viruses, bacteria, and parasites, are then discussed with relationship to their associated malignancies. Although not exhaustive, the epidemiology, clinical manifestations, pathogenesis, and histological findings are included, along with a short review of emerging therapies.

## 1. Introduction 

Cancer is the leading cause of premature death in 57 countries, along with cardiovascular diseases in 70 countries, and it is anticipated that cancer may supersede cardiovascular diseases as the leading cause of worldwide premature death in this century [1]. Based on the GLOBOCAN 2020 estimates, approximately 19.3 million cases of cancer were newly diagnosed in 2020, with mortality in 10.0 million cases [2]. It has been estimated that 35.0% of cancer deaths were attributable to potentially modifiable risk factors including smoking, alcohol use, low fruit and vegetable intake, overweight and obesity [3], with infections agents representing the third leading cause of cancer following smoking and diet [4]. 

Infection-attributable cancers account for 15.0% to 20.0% of the worldwide cancer burden [5,6,7]. Newer data reported 2.2 million cancer cases attributable to infection in 2018, with the highest incidence rates reported in infections with Helicobacter pylori (H. Pylori) (8.7 cases per 100,000 person-years), human papillomavirus (HPV) (8.0 cases per 100,000 person-years), hepatitis B virus (HBV) (4.1 cases per 100,000 person-years), and hepatitis C virus (HCV) (1.7 cases per 100,000 person-years) [8]. Incidence rates of these infection-attributable cancer cases were highest in eastern Asia and sub-Saharan Africa, with an incidence rate of 37.9 cases and 33.1 cases per 100,000 person-years, respectively [8]. Furthermore, there is significant economic burden associated with infection-attributable cancers. A Korean study reported direct costs of these cancers amounting to USD 676.9 million and indirect costs amounting to USD 2.57 billion in 2014, which accounted for 0.23% of the gross domestic product and 1.36% of the healthcare expenditure [9]. 

The International Agency for Research on Cancer (IARC) has classified ten infectious pathogens as carcinogenic to humans (group 1) which include H. Pylori, HBV, HCV, HPV, Epstein–Barr virus (EBV), human herpesvirus type 8 (HHV-8), human T-cell lymphotropic virus type 1 (HTLV-1), Opisthorcis viverrini (Ov), Clonorchis sinensis (Cs), and Schistosoma haematobium (Sh) [10]. Human immunodeficiency virus (HIV) will be discussed with the associated pathogens as it causes cancer through immunosuppression and is not oncogenic in itself [11]. Merkel cell polyomavirus (MCPyV) was also described to be implicated in merkel cell carcinoma in 2008 [12] and will be discussed. The cancers associated with these group 1 pathogens are often encountered in routine histo- and cytopathology practice. While they usually have characteristic morphologies when evaluated histologically or cytologically, ancillary immunochemical and special stains may also help to delineate the exact phenotype of these cancers. Benign mimickers of malignancies due to viral infections of the affected cells or granulomatous inflammation are also often encountered in diagnostic practice and this is especially pertinent in cytopathology. 

The emergence of the novel coronavirus SARS-CoV-2 (COVID-19) in late December 2019 has shifted much attention to controlling this global health crisis [13]. There is emerging evidence supporting the similarities between COVID-19 and cancers, such as the identification of oncogenic pathways targeted by SARS-CoV-2 [14,15]. Further, it has been reported that SARS-CoV-2 encoded proteins can induce the lytic reactivation of HHV-8 in latently infected cells [16]. As such, a comprehensive understanding of these oncogenic pathogens is warranted, given the current worldwide burden of COVID-19. 

**Figure 1:**
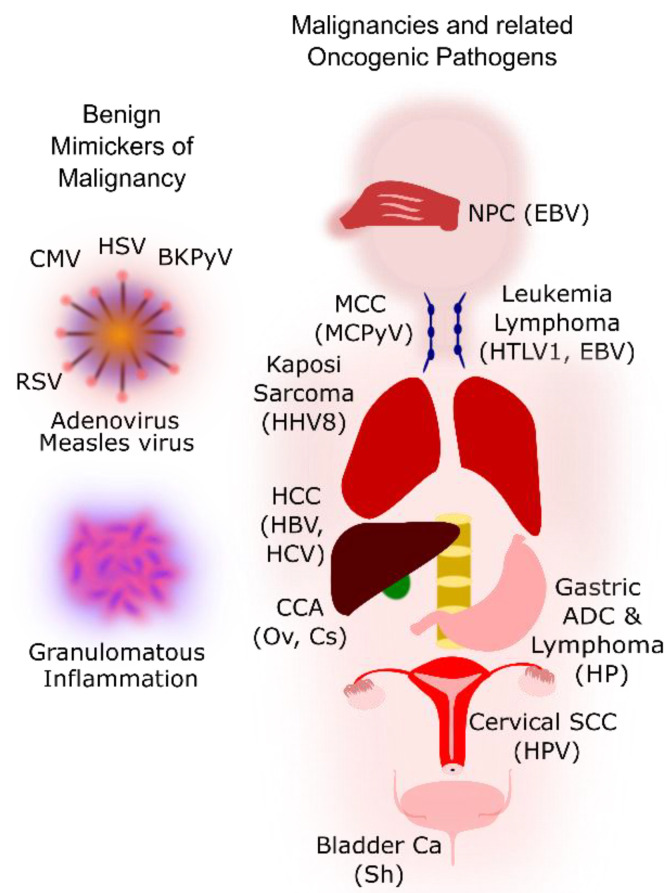
Outline of the benign mimickers of malignancy and the malignancies associated with oncogenic pathogens. CMV, cytomegalovirus; HSV, herpes simplex virus; BKPyV, BK polyomavirus; RSV, respiratory syncytial virus; NPC, nasopharyngeal carcinoma; EBV, Epstein-Barr virus; MCC, merkel cell carcinoma; MCPyV, Merkel cell polyomavirus; HTLV1, human T-lymphotropic virus type 1; HHV8, human herpesvirus 8; HCC, hepatocellular carcinoma; HBV, hepatitis B virus; HCV, hepatitis C virus; CCA, cholangiocarcinoma; Ov, Opisthorcis viverrini; Cs, Clonorchis sinensis; Sh, Schistosoma haematobium; Ca, carcinoma; ADC, adenocarcinoma; HP, Helicobacter pylori; SCC, squamous cell carcinoma; HPV, human papillomavirus.

Figure 1 shows some common benign mimickers of malignancy and the malignancies associated with the group 1 pathogens. Benign mimickers related to viral infections often show slightly atypical histological and cytological features such as enlarged nuclei, but these findings are well described in the literature and can be distinguished based on the presence of other benign features such as fine chromatin and smooth and regular nuclear contours. We first describe the benign histological and cytological changes seen with various pathogens and then discuss the malignancies associated with the oncogenic pathogens. Pertinent diagnostic conundrums associated with the benign mimickers of malignancy are emphasised. We also discuss relevant epidemiology, pathogenesis, clinical manifestations, and emerging management options including immunotherapy in the treatment of these malignancies.

## 2. Benign Mimickers of Malignancy

### 2.1. Herpes Simplex Virus (HSV)

HSV is a member of the Herpesviridae family of viruses which have enveloped double-stranded DNA genomes [17,18]. This family can be classified into three subfamilies by tissue tropism: alphaherpesviruses, which include herpes simplex virus 1 (HSV-1), herpes simplex virus 2 (HSV-2), and varicella zoster virus (VZV), that infect epithelial cells and remain latent in the neuronal cell body; betaherpesviruses, which include cytomegalovirus (described below), human herpesvirus-6, and human herpesvirus-7, that infect a variety of cell types; and gammaherpesviruses, which include Epstein–Barr virus and human herpesvirus-8 (both described below), that infect lymphoid and other cells [19]. A typical sequence of infection, namely, acute infection followed by latency and variable rounds of viral reactivation, is characteristic of herpesviruses. Approximately 40% to 98% of the general population possess antibodies to HSV-1 [17].

HSV1 is typically implicated in orofacial infections (for example, cold sore of the lip) and sporadic encephalitis, while HSV2 is typically implicated in genital infections (for example, vesicles and ulcers) and may be transmitted from mother to newborn [20]. As an aide memoire, HSV1 typically causes infections in the superior part of the body, while the reverse holds true for HSV2 (“1 is superior to 2”). Immunocompromised patients may be susceptible to disseminated disease including encephalitis and visceral organ involvement, such as the gastrointestinal and respiratory systems, resulting in clinical manifestations like pneumonia. Similarly, maternal-neonatal transmission, although rare, is associated with disseminated disease, encephalitis, thrombocytopenia, and disseminated intravascular coagulation in the neonate. Foetal infection may result in microcephaly, retinitis, and scarring of the skin [20]. 

**Figure 2:**
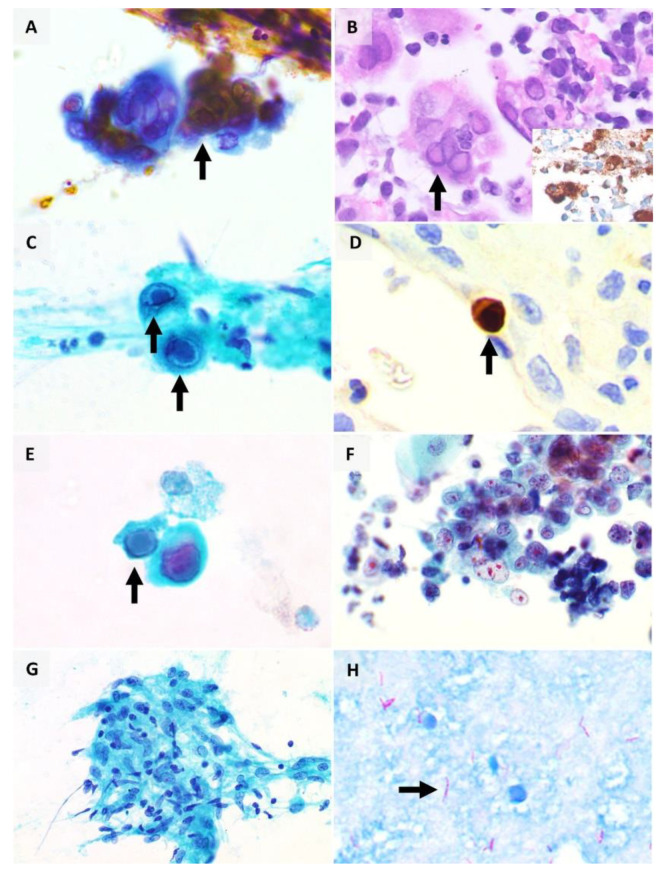
Benign mimickers of malignancy. (**A**) HSV viral cytopathic effect on cytology (arrow), with intranuclear inclusions and nuclear moulding; (**B**) HSV viral cytopathic effect (arrow) on formalin-fixed paraffin-embedded (FFPE) tissue (inset: immunohistochemistry for HSV antigen); (**C**) CMV viral cytopathic effect on cytology, with characteristic Owl’s eye nuclear inclusions (arrows); (**D**) CMV immunohistochemistry (arrow) on FFPE tissue; (**E**) BKPyV viral cytopathic effect, also termed ‘decoy cells’ (arrow), showing glassy basophilic nuclear inclusions; (**F**) cytology of high-grade urothelial carcinoma, which is an important differential diagnosis of decoy cells; (**G**) granulomatous inflammation, consisting of clusters of epithelioid histiocytes; (**H**) Ziehl–Neelsen stain for acid-fast bacilli (arrow).

In modern medicine, microbiologic confirmation can be obtained by polymerase chain reaction (PCR) or viral isolation [17]. HSV viral cytopathic effect is seen in approximately 0.15% to 0.24% of cytology specimens including vaginal smears and respiratory specimens such as sputum, bronchoalveolar lavage, bronchial washings, and brushings [21,22]. The distinctive cytomorphologic features (Figure 2A,B) can be summed up by the “3Ms” — margination of chromatin (due to intranuclear viral inclusions that push the host cell chromatin to the periphery of the cell nucleus), multinucleation (due to fusion of cells), and nuclear moulding (conformity of adjacent nuclei) [18]. The chromatin appearance is often likened to a ground-glass smudged appearance (termed ‘Cowdry type B’ inclusions) while eosinophilic intranuclear inclusions rimmed by a clear halo (termed ‘Cowdry type A’ inclusions) can also be seen [18]. These features are typically seen in epithelial cells, such as the squamous cells of the epidermis or mucosa. VZV-infected cells also show similar appearance to that seen in HSV [17,23]. Adjacent acute inflammation and epithelial ulceration may also be seen.

HSV viral cytopathic infection can mimic malignancy on cytology. In a retrospective study of 18 cases of respiratory cytological specimens with the diagnosis of HSV infection, only 28% had classic viral cytopathic changes while 22% showed atypical squamous cells with dense keratinised cytoplasm, hyperchromatic nuclei with irregular contours, features which can be mistaken for a squamous cell carcinoma (SCC) [22]. It is also reported that HSV-infected respiratory epithelial cells may show a less typical viral cytopathic effect compared to that seen in other sites, such as a lower tendency to exhibit multinucleation [22].

Although HSV antigens can be demonstrated on cytological smears such as cervical smears [24] and immunohistochemistry (Figure 2B inset) for HSV is available as an ancillary diagnostic tool, it is not routinely required given the distinctive morphologic features [25]. Treatment for HSV infections includes anti-viral agents, such as acyclovir and valaciclovir. As expected, the dose, route and duration of therapy are contingent on the site and severity of infection as well as the host immune system [20].

### 2.2. Cytomegalovirus (CMV)

CMV is an enveloped double-stranded DNA virus [26,27], a member of the betaherpesviruses subfamily [28]. It is ubiquitous in humans and acquired by most in early life [29]. Reports suggest that between half to all adults possess the IgG antibodies to CMV, indicating previous exposure [19]. Inhibition of viral lytic gene expression is a key driver of latency [30], and the virus may remain latent in a diverse range of cells including endothelial cells, epithelial cells, stromal cells, and lymphocytes. Subsequent reactivation is thought to occur when the balance between viral load and host immune system causes a “threshold” to be exceeded [30].

As with other infections, the clinical presentation of CMV infection depends upon the host immune status. In immunocompetent individuals, acute infection may be asymptomatic or may present as self-limiting, non-specific febrile illness or as mononucleosis-like illness [17]. In contrast, immunocompromised hosts are susceptible to systemic infections, such as CMV infection of the respiratory tract resulting in manifestations like interstitial pneumonitis [17,18], and those involving the gastrointestinal tract, liver, and the retina [28]. CMV has also been reported in cervical smears [31]. CMV pneumonitis is associated with a high mortality rate of 30% to 50% [17]. Due to the acuity of CMV pneumonitis, respiratory specimens such as bronchoalveolar lavage (BAL) [17,32,33,34] or bronchial washings [35] are often obtained for diagnosis. 

Diagnosis can be achieved by molecular-based methods such as PCR or non-molecular based methods such as serology, detection of antigen, and histopathologic examination [29,36]. PCR was reported to be more sensitive but less specific than viral culture [17]. In a series of 11 BAL samples with CMV isolated in culture, cytology had a sensitivity of 73% and specificity of 100%, which was higher when compared to in situ hybridisation (sensitivity of 55% and specificity of 94%) [34].

On histology, infected cells are typically identifiable at low power (as a result of cytomegaly) and bear enlarged nuclei (nucleomegaly). The cells have characteristic Owl’s eye nuclear inclusions (represented by viral particles surrounded by a clear halo due to shrinkage of the viral particles from the nuclear membrane after tissue fixation) (Figure 2C) and intracytoplasmic inclusions [18]. These inclusions have been reportedly seen in the respiratory epithelium, pneumocytes, macrophages, endothelium, and interstitial cells [18]. The cytomorphologic appearance of CMV-infected cells in both conventional cytologic smears and liquid-based cytology was investigated in a series of five bronchial washings [37], where the authors reported that intranuclear inclusions, size of cytoplasm and nucleus appeared larger in liquid-based cytology than in conventional smears but the nucleus-to-cytoplasm ratio was larger in conventional smears, which could be mistaken for atypia or malignancy [37]. 

Like HSV, immunohistochemistry for CMV (Figure 2D) is also available as an ancillary diagnostic tool. In a case series of 17 cases with CMV-positive transbronchial biopsy and CMV-negative bronchoalveolar lavage, the use of immunocytochemistry for detection of CMV improved the sensitivity of cytologic detection of CMV [38]. 

Treatment of CMV infection is conventionally achieved with anti-viral agents such as ganciclovir and valganciclovir, on a regime that is guided by the severity of infection, host immune status and the viral response over time [29]. Immunocompromised patients may be eligible for anti-viral prophylaxis. Given its latency and pervasiveness, the long-term effect of latent CMV infection is a matter of great research interest. It has been indirectly linked to mortality by way of increase in activated T cells leading to inflammation and cardiovascular disease, as well as reduction in naïve T cells in the elderly, rendering them less responsive to routine vaccination such as influenza and pneumococcal vaccines [30]. 

### 2.3. BK Polyomavirus (BKPyV)

Human Polyoma Virus 1, more commonly known as BK polyoma virus (BKPyV) after the initials of the first affected patient, is a member of the polyomaviridae family. It is a non-enveloped virus with a double-stranded DNA genome [39]. Like CMV, it is a relatively ubiquitous pathogen, with more than 90% of the population acquiring primary infection in childhood [40]. This virus has a tropism for the urological system and remains latent in the renal tubular epithelium and urothelium [41]. Clinically significant reactivation occurs almost exclusively in the immunocompromised [39], namely, in renal transplant patients who may develop polyomavirus-associated nephropathy (PyVAN) and in haematopoietic stem cell transplant (HSCT) patients who may develop polyomavirus-associated haemorrhagic cystitis (PyVHC). In contrast, only minority of immunocompetent hosts develop asymptomatic viruria. BKPyV is also implicated in other organ systems, presenting as encephalitis, meningitis, pneumonia, retinitis, colitis, and vasculitis [39,42], amongst other manifestations such as upper respiratory disease and tonsillitis [43]. PCR is most often used for diagnosis of BKPyV, followed by viral culture and serology [39].

Histologically, BKPyVAN progresses from an early state of viral cytopathic effect and acute tubular injury (pattern A) to interstitial nephritis (pattern B) and finally to severe interstitial fibrosis (pattern C) [44]. Viral cytopathic effect is typified by renal tubular cells with enlarged nuclei with glassy basophilic nuclear inclusions [39]. This can be confirmed via immunohistochemistry using monoclonal antibodies against Simian polyomavirus SV40, which cross-react with BKPyV. As BKPyVAN is a significant cause of allograft failure, early recognition of the disease process is key to improving patient outcomes. The mainstay of treatment is to reduce immunosuppression which can be achieved by reducing drug dose, stopping or switching drugs [41]. 

In the bladder, urothelial cells infected by BKPyV similarly show enlarged nuclei with glassy basophilic nuclear inclusions and are termed ‘decoy cells’, (Figure 2E) as they may be mistaken for malignant urothelial cells (Figure 2F) on cytologic preparations. Absence of other cytologic features of malignancy, such as coarse chromatin and irregular nuclear contours as well as correlation with the clinical and cystoscopic findings are pertinent considerations in distinguishing decoy cells from malignant urothelial cells [45]. Although BKPyV can be detected in urine cytology, urine PCR has been reported to have higher sensitivity and specificity compared to cytology [46]. Other methods of BKPyV detection also include immunofluorescence microscopy and electron microscopy [47]. 

In a study of 240 urine cytology specimens from 80 renal transplant recipients [48], decoy cells were identified in 37.5% and has been proposed as a routine screening method for viral infections in renal transplant patients. This is further supported by another study of 13 patients with BKPyV found in urine that reported a positive predictive value for polyomavirus disease of 90% [49].

The relationship between BKPyV with autoimmune diseases such as systemic lupus erythematosus [42] and malignancy is controversial and the pathogenesis is thought to be independent of active viral replication [41]. BKPyV has been shown to induce the transformation of normal cells into malignant cells, partly attributed to the expression of major Tumour-Antigen and minor Tumour-Antigen [39]. In a study investigating the association between BKPyV DNA, mRNA, and cancers, including renal cell carcinoma and bladder transitional cell carcinoma [50], there was a significant association between the presence of BKPyV DNA and renal cell carcinoma, with an increase in risk of developing renal cell carcinoma in patients infected with BKPyV. Other studies have reported an association between BKPyV and urothelial carcinoma in both immunocompromised and immunocompetent patients [40,51]. BKPyV is also associated with prostatic carcinoma [51,52,53]. However, several studies have reported a lack of association between BKPyV and urothelial carcinoma, where one study reported a lack of BKPyV DNA sequences in transitional cell carcinomas of the bladder [54] and another retrospective study of 37 cases of urothelial carcinomas did not show evidence of BKPyV on immunohistochemistry and chromogenic in situ hybridisation [55]. Further studies are warranted in this area.

### 2.4. Adenovirus, Measles Virus, and Respiratory Syncytial Virus (RSV)

Adenovirus, a non-enveloped icosahedral virus with a DNA-protein core complex [56], is subdivided into multiple subgroups ranging from subgroup A to F, each made up of different serotypes and having a predilection for specific organs including the gastrointestinal, respiratory, and urinary tracts [57]. Subclinical infections often occur in immunocompetent hosts and clinically significant manifestations include upper and lower respiratory tract disease, conjunctivitis, gastroenteritis, and cystitis [56]. Adenovirus can be detected in conjunctival scrapings, nasopharyngeal aspirates, urine, and stool samples via PCR, cell culture, and antigen detection by immunofluorescence [58,59]. Viral cytopathic changes associated with adenovirus can mimic malignancy in cytology specimens including pleural fluid [60] and urine samples [61], and these changes typically include enlarged nuclei with basophilic intranuclear inclusions surrounded by a thin rim of cytoplasm [60], also termed ‘smudge cells’ [17]. Adenovirus infection is often managed supportively although antivirals such as cidofovir and ribavirin have been explored in the treatment [56]. 

Measles is an enveloped spherical RNA virus [62] which was notable for its high mortality rate of up to millions of deaths each year prior to the introduction of vaccination [62]. The initiation begins with infection of the respiratory epithelium of the host [63,64], followed by an incubation period of 5 to 11 days [63,64], which then leads to the symptomatic stage where clinical manifestations including fever, malaise, conjunctivitis, as well as the characteristic Koplik’s spots (white spots on buccal mucosa) [64,65]. Measles can also present as a systemic disease involving other organ systems [63]. The diagnosis of measles can be confirmed using serologic tests, cultures, and PCR [65]. Eosinophilic cytoplasmic and intranuclear inclusion bodies are frequently reported in patients with measles, where these inclusions have been reported to occur in the skin, respiratory tract, urinary tract [64,66,67], and central nervous system [63]. Types of specimens include sputum, nasal secretions, and upper respiratory tract swabbing, and BAL [17]. In addition to inclusion bodies, multinucleated giant cells with overlapping nuclei and variation in shape and size [17] are also frequently observed [64,67], postulated to occur due to the fusion of type 2 pneumocytes [17]. These cytologic features, although non-specific, may mimic giant cell rich malignancies. The management of measles infection is also primarily supportive and antivirals have also been used to treat severe measles [62].

Primarily causing illness in infants and elderly people [68], RSV is an enveloped RNA virus [18] that infects the upper respiratory tract and eyes [68]. RSV infection is clinically apparent with upper respiratory tract signs and symptoms including cough, wheezing, and low grade fever [69], as well as other manifestations including otitis media, bronchiolitis, and pneumonia, which usually happen in children [68]. Depending on the clinical manifestation, radiologic findings may show typical findings such as hyperinflation, diffuse interstitial markings and peribronchial thickening [68]. The diagnosis of RSV is established through detection of RSV antigens in nasopharyngeal aspirates through immunofluorescence, enzyme-linked immunosorbent assay (ELISA) or culture [68]. Characteristic cytological findings have been described in RSV infections, including presence of large syncytial cell aggregates with eosinophilic cytoplasmic inclusions [18]. Creola bodies, which are clusters of reactive ciliated bronchial epithelial cells, have also been reported in RSV bronchiolitis [18]. These clusters of Creola bodies may be confused with adenocarcinoma on cytology but the presence of cilia reinforces the benign nature of these cells. Treatment in RSV infection is symptomatic management and aerosolised ribavirin is approved for use in hospitalised infants [70]. 

### 2.5. Granulomatous Inflammation

Granulomatous inflammation is defined by the presence of granulomas, which refer to aggregates of epithelioid histiocytes (Figure 2G) [71]. Granulomatous inflammation has been extensively discussed in previously published reviews [17,71,72,73]. Several patterns of granulomatous inflammation have been described, and they include foreign body type reaction, necrotising (with central necrotic material) granulomas, and non-necrotising granulomas [71]. Different patterns of granulomatous inflammation are associated with different aetiologies. Foreign body type reaction is often associated with suture material in excision or resection specimens, as well as other foreign materials such as talc and starch [71]. Necrotising granulomas are associated with infectious pathogens including mycobacterium tuberculosis and fungal organisms [17,71], while the differential diagnoses of non-necrotising granulomas encompass both infectious and non-infectious causes including sarcoidosis [74], Crohn’s disease [75], toxic and drug causes [71]. The Ziehl–Neelsen stain (Figure 2H) is often used clinically to identify acid-fast bacilli in respiratory specimens including sputum, with a specificity ranging from 90% to 100%, albeit with a limited sensitivity [17]. 

The presence of granulomas associated with tumours is well established, reportedly seen in 4.4% of carcinomas, 13.8% of Hodgkin’s disease, 7.3% of non-Hodgkin lymphomas [76], and 50.0% of seminomas [77,78]. Distinguishing between a granulomatous inflammation and malignancy has proven to be difficult on fine needle aspiration cytology (FNAC) [78], where a study of six cases of neck mass FNAC reported that only one case was diagnosed as metastatic carcinoma with extensive granulomatous inflammation while the remaining cases were signed out as “atypical” with a recommendation for tissue biopsy [78]. The histopathological diagnoses of the remaining cases included Hodgkin’s disease, lymphoepithelial carcinoma, diffuse large B-cell lymphoma, and anaplastic carcinoma [78]. In a subsequent study of 153 patients undergoing endobronchial ultrasound-guided transbronchial needle aspiration for mediastinal lymphadenopathy which met radiological criteria for cancer recurrence, 11.0% showed non-caseating granulomas on cytology [79]. Granulomatous inflammation also occurs after intravesical Bacillus Calmette-Guerin therapy for bladder carcinoma [80].

In particular, granulomatous mastitis, a granulomatous inflammation of breast parenchymal tissue, although uncommonly encountered, can be mistaken for a malignancy, both clinically and radiologically [81]. A series of three studies of patients with granulomatous mastitis reported a mean age of 34 to 44 years [81,82,83]. In one of these studies, tuberculous infection accounted for most cases of granulomatous mastitis, followed by foreign body and idiopathic aetiologies [82]. Of these three studies, one was a series of 16 patients which reported clinical impression of a breast abscess in half the patients [81], whereas another series of 18 cases [83] reported a clinical impression of a malignant lesion in all cases. The most common ultrasonography finding in these studies was a heterogeneous hypoechoic lesion [82]. Cytologic and histological findings were characteristic for granulomas, lymphocytes, and plasma cells, but differed in the presence or absence of necrosis [82]. An early, limited case series of five patients with granulomatous mastitis [84] reported atypia or ‘suspicious for malignancy’ on FNAC in three cases. However, the authors did not specify the criteria used for a cytologic impression of an ‘atypical’ lesion. There is now a trend towards performing core needle biopsy over FNAC in sampling breast lesions [85], thereby improving diagnostic accuracy by histopathology rather than cytology.

## 3. Oncogenic Pathogens

### 3.1. Hepatitis B and C Viruses (HBV, HCV) 

HBV and HCV are hepatotropic viruses implicated in acute hepatitis, chronic liver disease, and cirrhosis as well as hepatocellular carcinoma (HCC). 

Hepatitis B is a member of the Hepadnaviridae family of viruses. It bears a double-stranded DNA genome, which is able to integrate into the DNA of the host genome. There are 10 major genotypes of which genotype C and D are associated with a higher risk of progression to liver cirrhosis [86]. In childhood, the majority of infections acquired tend to persist as chronic infections, while the reverse holds true for infections acquired in adults, in whom the majority are able to clear the infection [87]. 

On the other hand, Hepatitis C is a member of the Flaviviridae family of viruses. It bears a single-stranded RNA genome. There are seven major genotypes of which genotype 1 is associated with more aggressive disease course and reduced responsiveness to therapy. Once acquired, Hepatitis C persists as a chronic infection in approximately 80% of hosts [86]. 

**Figure 3:**
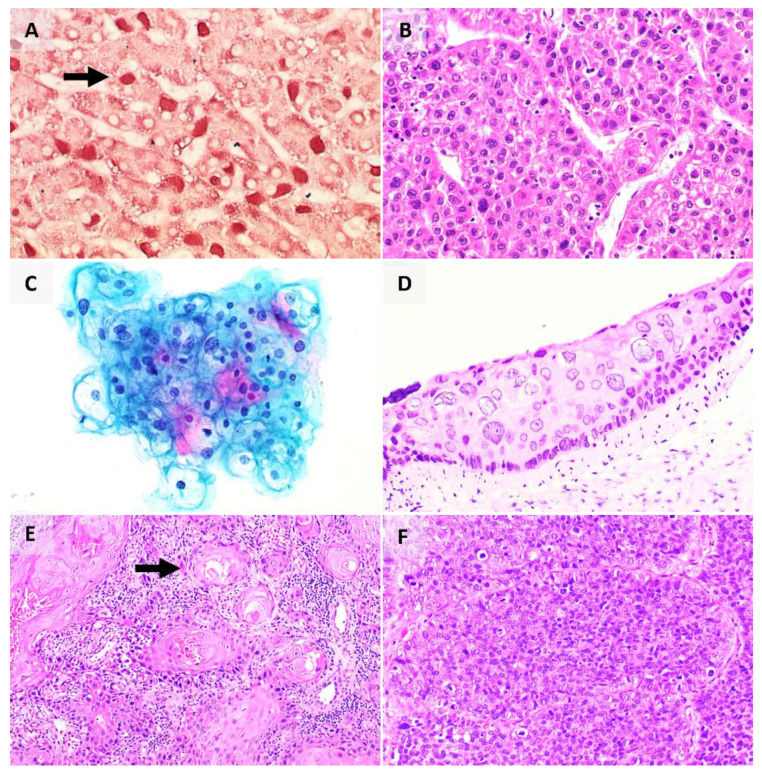
(**A**) HBV surface antigen highlighted by orcein stain (arrow); (**B**) HCC, with expanded hepatic plates and nuclear atypia; (**C**) HPV effect seen on cytology, characterised by cytoplasmic perinuclear cavitation; (**D**) HPV effect seen on FFPE tissue; (**E**) keratinising SCC, characterised by formation of keratin pearls (arrow) and proliferation of malignant squamous cells with enlarged and hyperchromatic nuclei; (**F**) non-keratinising SCC showing large islands and nests of ovoid to spindle-shaped cells with hyperchromatic nuclei, without formation of keratin pearls.

Clinical diagnosis of Hepatitis B or Hepatitis C infection can be achieved via serology or molecular methods. The role of histopathologic examination is primarily in the grading and staging of liver disease. Of note, the accumulation of Hepatitis B surface antigen (HBsAg) in hepatocytes gives a ground glass appearance to the cytoplasm which stains magenta and blue on interrogation with the Shikata’s orcein stain (Figure 3A) and Victoria blue stain, respectively. The other features of active or chronic liver disease in Hepatitis B may, to some extent, overlap with other conditions [87]. In contrast, Hepatitis C infection in the liver is associated with a hallmark triad of bile duct injury, lymphoid follicles with germinal centres, and small and large droplet steatosis. 

Approximately 70–85% of cases of HCC worldwide are associated with Hepatitis B and Hepatitis C infections [19]. Despite this, its oncogenesis remains incompletely elucidated. While many factors are implicated, the major player in the oncogenic process is believed to be virally induced inflammation that leads to hepatocyte injury, proliferation, regeneration and eventually accumulation of genomic damage [19]. In most instances, the diagnosis of HCC can reliably be made on its characteristic radiologic appearance as a lesion with arterial enhancement and early portal venous washout, reflecting its predominantly arterial blood supply against the background liver which receives most of its blood supply from the portal veins. Histopathologic examination is typically applied to resection specimens for the pathological staging of the carcinoma. Histologically, HCC shows four morphologic patterns including trabecular, solid, pseudoglandular, and macrotrabecular growth patterns, made up of tumour cells showing characteristic changes such as bile production and fatty change (Figure 3B) [88]. HCC shows immunostaining for hepatocytic markers including HepPar-1 and arginase-1 [88].

The management of HCC includes surgical resection, chemoembolisation, radioembolisation, and liver transplantation [89]. Sorafenib is approved for treatment for advanced HCC [90]. Immunotherapy with agents such as nivolumab and pembrolizumab is also quickly gaining recognition as showing promising results in HCC therapy [90,91]. The use of neoantigen vaccine is emerging as a potential immunotherapy in HCC [92]. Neoantigens are proteins that are produced during cellular events such as gene mutation and they are immunogenic [92]. It has also been postulated that HBV-related HCC possesses more effective neoantigens compared to non-HBV-related HCC, and this may be a potential novel target for vaccines in the future [92]. 

### 3.2. Human Papillomavirus (HPV)

Human papillomavirus (HPV) is a member of the Papovaviridae family. It is a non-enveloped DNA virus with a predilection for cutaneous and mucosal epithelium. More than 90% of people who acquire the virus experience transient infection and can immunologically clear the virus. In contrast, chronic persistent infection, contributed to by various factors such as immunocompromise, frequent reinfections and co-infections, is associated with progression to neoplasia [93]. HIV status was reported to be associated with higher prevalence of HPV infection [94].

More than 100 types of HPV virus have been isolated and sequenced to date. These can be broadly subclassified into low-risk and high-risk types, based on malignant potential. Low-risk types include HPV 6 and 11 which are associated with genital warts and respiratory papillomatosis. High-risk types include HPV 16, 18, 31, 33, 45, and 51, which are associated with malignancies of the cervix, anogenital region, and oral cavity. The central oncogenic event is the integration of the viral genome into the host genome, which results in the loss of the viral E2 repressor leading to increased expression of oncogenic proteins E6 and E7 that inhibit p53 and Rb proteins, respectively [19]. 

In the cervix, the neoplastic process is represented by the progression across well-defined morphologic categories of cervical intraepithelial neoplasia (CIN) 1, 2, and 3, and finally invasive SCC. The hallmark of HPV infection is koilocytes (hollow cell), which are mature squamous cells that show binucleation, nuclear hyperchromasia, nuclear contour irregularities and enlargement, as well as the characteristic perinuclear halo (due to interaction between the viral E4 protein and cytokeratin filaments causing their shift away from the nucleus) that gives the cell its name (Figure 3C,D) [95]. Histologically, HPV-associated SCC (Figure 3E,F) shows a proliferation of squamous cells, with markedly pleomorphic nuclei, and showing features of squamous differentiation such as intercellular bridges [96]. They may show distinctive growth patterns including basaloid, condylomatous, and papillary patterns [96]. These tumours show positive staining for p16 on immunohistochemistry [96].

Remarkably, our understanding of the role of HPV in cancer continues to evolve, with the recognition of new entities such as HPV-related multiphenotypic sinonasal carcinoma (associated with HPV type 33) [97], and the reclassification of established entities, such as cervical adenocarcinomas that are now formally classified by HPV status (HPV-associated or HPV-independent), as per the latest iteration of the World Health Organization classification of tumours of the female genital tract [96]. 

Public health measures such as cervical cancer screening programmes using Papanicolaou smears, liquid-based samples or primary HPV testing by molecular methods, and universal HPV vaccination have seen great success in developed countries which have witnessed a decline in rates of squamous cell carcinoma of the cervix over the years. These public health measures hold great promise in less developed populations that continue to suffer from relatively high rates of SCC of the cervix, an increasingly preventable cancer. 

The management of cervical cancers depends on the stage of disease, with options including radical surgery and radiotherapy, as well as neoadjuvant chemoradiotherapy [98]. Immunotherapy is currently being explored in treating HPV-associated malignancies [99], and options include the use of immune checkpoint inhibitors and therapeutic vaccines [99]. Therapeutic vaccines are postulated to incite inflammatory responses against the viral proteins E6 and E7 [100,101]. 

### 3.3. Epstein–Barr Virus (EBV)

Epstein–Barr Virus (EBV) is a member of the gammaherpesviruses subfamily. It is a ubiquitous virus acquired by most people by young adulthood. The virus is transmitted by saliva and infects epithelial cells and B cells of the oropharynx, causing a predominantly lytic infection in the former while establishing latency in the latter [102]. 

In immunocompetent hosts, primary infection is typically asymptomatic or presents as infectious mononucleosis, a classic constellation of fever, tonsillitis/pharyngitis, lymphadenopathy, and atypical lymphocytosis. The diagnosis can usually be made on clinical grounds supported by serologic confirmation. Rarely, atypical clinical presentation leads to consideration of biopsy of the enlarged tonsil or lymph node [103]. Interpretation of such biopsies is challenging because infectious mononucleosis may manifest as an atypical lymphoid infiltrate with architectural distortion, immunoblastic proliferation, and Reed Sternberg-like cells, thus mimicking lymphoma [104]. The polyclonal B cell lymphoproliferation is typically curtailed by a robust T cell response in an immunocompetent host. 

**Figure 4:**
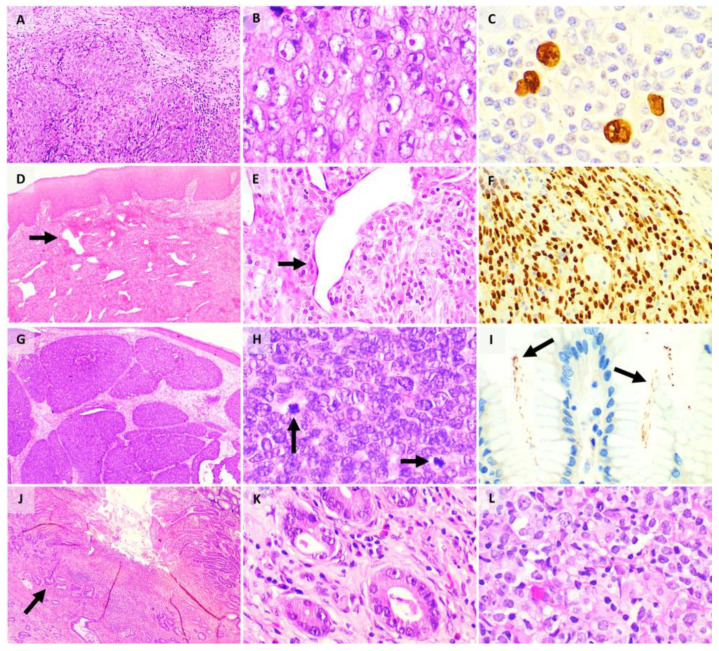
(**A**) Low power magnification of non-keratinising NPC, made up of a syncytial arrangement of cells; (**B**) high power magnification of NPC, with cells showing round nuclei, prominent eosinophilic nucleoli, within an eosinophilic to amphophilic cytoplasm; (**C**) EBV-encoded RNA in-situ hybridisation for detection of EBV; (**D**) low power magnification of KS, showing slit-like vascular spaces (arrow) in the dermis of the skin; (**E**) high power magnification of KS, showing vascular spaces (arrow) lined by cells with hyperchromatic nuclei; (**F**) HHV-8 immunostain, showing nuclear positivity; (**G**) low power magnification of MCC, made up of islands of “blue cells” in the dermis of the skin; (**H**) high power magnification of MCC, showing cells with increased nucleus/cytoplasm ratio, salt-and-pepper chromatin, and frequent mitoses (arrows); (**I**) Identification of HP (arrows) by immunohistochemistry; (**J**) low power magnification of gastric adenocarcinoma (arrow), consisting of infiltrative glands in the stomach wall; (**K**) high power magnification of gastric adenocarcinoma, with glands made up of cells with enlarged nuclei, and prominent nucleoli; (**L**) gastric DLBCL, consisting of high-grade lymphoid cells.

In the immunocompromised, such as in a patient with HIV, however, an uncontrolled B cell lymphoproliferation results, leading to acquisition of additional genomic alterations culminating in a monoclonal proliferation that amounts to lymphoma [19,105]. Broadly, these are termed EBV-associated lymphoproliferative diseases (including B cell lymphomas and NK/T cell lymphomas). Epithelial malignancies such as nasopharyngeal carcinoma (NPC) (Figure 4A,B) and lymphoepithelial carcinoma of various primary sites and mesenchymal tumours like EBV positive smooth muscle tumours are also well-documented EBV-associated malignancies, reflecting the virus’s diverse oncogenic potential. On tissue sections, the EBV status of a tumour can be interrogated by Epstein–Barr-encoded RNA in situ hybridisation (EBER-ISH) (Figure 4C).

The development of prophylactic EBV vaccines for young children (prior to acquisition of the virus) or in pre-transplant patients is a subject of ongoing research [104]. Therapeutic vaccines have also been primarily studied in NPC and have shown promising early results [104,106]. Other immunotherapies are also currently explored in EBV-associated NPC [106].

### 3.4. Human Herpes Virus 8 (HHV8)

HHV8, also termed Kaposi sarcoma (KS)-associated herpes virus, belongs to the gammaherpesvirus subfamily and is a large double-stranded DNA within an enveloped capsid [107]. It is associated with KS, primary effusion lymphoma (PEL), multicentric Castleman disease (MCD), HHV-8 positive diffuse large B-cell lymphoma (DLBCL), and germinotropic lymphoproliferative disorder (GLPD), and these disorders, with the exception of GLPD, are frequently associated with HIV infection [108]. 

The incidence of KS is highest in Central Africa [107]. The epidemiology of HHV8 can be delineated based on the variants of KS, namely, classic, endemic (African), transplantation-associated (iatrogenic), and epidemic (AIDS associated) [109]. Classic KS occurs mainly in elderly men of Mediterranean, Eastern European, Jewish, and South American descent [107,109,110] while endemic KS occurs frequently in certain Central African and sub-Saharan African countries [109,110]. Iatrogenic KS occurs in up to 5% of transplant recipients [109] and immunocompromised patients [110] while epidemic KS is currently the most prevalent form of KS, affecting mainly homosexual males [109]. The prevalence of HHV8 infection is estimated to be less than 3% to 10% in the United States of America, United Kingdom, and Europe, while it ranges from 4% to 35% in Mediterranean countries like Italy and Greece and 30% to 60% in Africa [111]. 

The virus is transmitted primarily through sexual contact, especially through male homosexual contact, with other routes of transmission including mother-to-child, saliva, organ transplantation and other unknown routes [109,112]. The clinical manifestations of HHV8 infection are non-specific and can include fever, maculopapular rash, upper respiratory tract symptoms, diarrhoea, fatigue, and lymphadenopathy [113]. Classic KS manifests as bluish-red rashes on the distal lower extremities, which may eventually form multifocal nodules while the endemic KS has four clinical phenotypes, namely, nodular, florid, infiltrative, and lymphadenopathic [114]. Iatrogenic KS resembles classic KS on examination and epidemic KS often presents first on the nose, eyelids, ears, and the trunk [114]. PEL often presents as a lymphomatous proliferation involving the pleura, peritoneum and/or pericardium, typically without discrete masses [109]. HHV-8 is diagnosed based on serologic assays including immunofluorescence, ELISA, and Western blot while in situ hybridisation, PCR, and immunohistochemistry can be performed on tissues [109,113,115].

The pathogenesis and oncogenesis of HHV8 in the associated malignancies have been extensively reviewed [112,116,117,118]. HHV8 is postulated to enter cells mainly through the endocytic pathway and can infect various cell types including endothelial cells, and inflammatory cells [117]. The pathogenesis of HHV-8 involves latent and lytic gene expression [112,117,118]. In latent gene expression, the expression of latency associated nuclear antigen (LANA-1) amongst several other genes, is implicated in encouraging cell cycle progression and halting apoptosis in the malignancies associated with HHV-8 [118]. Lytic replication of the virus occurs in a small proportion of affected cells, resulting in production of mature virus [116], and the lytic genes include growth promoting genes like v-IL6 amongst numerous others [112,116,118]. It is postulated that both a ‘direct’ mechanism involving malignant transformation of benign endothelial cells and an ‘indirect’ mechanism involving the release of growth factors and cytokines could contribute to pathogenesis and oncogenesis [112].

Histologically, the various KS subtypes show an identical morphologic appearance. In the patch stage, there is a proliferation of vascular spaces in the upper reticular dermis, with flattened endothelial cells [119] (Figure 4D,E). In the plaque stage, there is more extensive proliferation of vessels, with jagged vascular spaces associated with a denser inflammatory infiltrate [119]. The nodular stage shows a circumscribed nodular proliferation of spindle cells arranged in fascicles [119]. On immunohistochemistry, the endothelial cells of intralesional vessels and lesional tumour cells are highlighted by endothelial markers including CD31, CD34, and ERG, and show nuclear positivity for HHV-8 [119,120,121] (Figure 4F).

PEL is a large B-cell neoplasm which shows a spectrum of morphological appearances ranging from immunoblastic or plasmablastic to anaplastic appearance, with large nuclei and prominent nucleoli within an abundant basophilic cytoplasm [108]. Some cells may appear similar to Reed–Sternberg cells seen in Hodgkin’s lymphoma, with brisk mitotic activity [108]. The lesional cells are typically positive for CD45 but negative for B-cell markers including CD19, CD20, CD79a, and PAX-5 [122]. There is nuclear positivity for LANA1 [108]. In MCD, the lymph node and splenic follicles show germinal centres with prominent mantle zones; “widened concentric rings” of lymphocytes and “prominent penetrating venules” may be seen, and this is typical of MCD (also termed ‘onion skinning’) [108]. HHV-8 positive DLBCL shows an effacement of lymphoid architecture, contributed by the “expansion of small confluent sheets of LANA1-positive plasmablasts” [108], while GLPD shows a preservation of architecture, with a proliferation of medium- to large-sized plasmablasts-like lymphoid cells [108]. The immunophenotype of MCD, HHV-8 DLBCL, and GLPD are reviewed elsewhere [108,122].

Chemotherapy with anthracyclines and taxols is the mainstay of treatment for KS [117]. Multiple medical therapies have been explored in managing HHV-8 infection and associated malignancies. Ganciclovir, a HHV-8 DNA synthesis inhibitor, has been proven to suppress HHV-8 replication and prevent development of KS [123], so its role is primarily preventative as opposed to therapeutic. Antiretroviral therapy (ART) has also been proven to slow down the rates of KS, such as with the use of nucleoside reverse transcriptase inhibitors (NRTI) and a non-NRTI or a protease inhibitor [123]. Immunotherapy has been shown to be a promising therapeutic option in HHV-8 related malignancies [124]. Programmed cell death ligand 1 (PD-L1) was reported to be expressed in 36.6% of classic KS and 28.6% of epidemic KS [125]. There have been ongoing trials investigating the use of nivolumab in cutaneous KS and pembrolizumab in KS [124].

### 3.5. Human T-Cell Leukemia Virus Type 1 (HTLV-1)

HTLV-1 was the first human retrovirus to be discovered, and consists of an enveloped single-stranded RNA [126]. HTLV-1 infection is endemic in the Caribbean, Africa, southwestern Japan, Italy, Middle East, South American, the Pacific Melanesian islands, and Papua New Guinea [126,127]. A review of 17 studies reported a prevalence of 36.4% in Japan, followed by 8.5% in Gabon, and 6.6% in Africa, with lowest prevalence rates reported in Mongolia, Malaysia, and India [128]. The primary mode of transmission of the virus is perinatally through breastfeeding, parenterally through blood transfusions or needle exposures, and sexually [126]. HTLV-1 infection can be detected with the use of ELISA to detect serum antibodies to core, envelop, and tax proteins, as well as Western blot assays and PCR [127]. The pathogenesis and oncogenesis of HTLV-1 are extensively reviewed elsewhere [129,130,131,132,133]. Also presented are the HTLV-1 gene codes for multiple structural proteins including Gag, Pol, and Env, and regulatory proteins like Tax, which activate viral replication and induce the expression of genes responsible for proliferation and anti-apoptosis of ATL cells [133]. The immortalisation of T cells is implicated in the oncogenesis of HTLV-1 [129]. 

HTLV-1 infection is associated with adult T-cell leukemia/lymphoma (ATL) and benign entities such as HTLV-1 associated myelopathy/spastic paraparesis [131]. There are four subtypes of ATL, namely, smouldering, chronic, acute, and lymphomatous [108]. Histologically, ATL shows a spectrum of morphological appearances, including a leukaemic pattern of proliferation, made up of medium- to large-sized lymphoid cells showing pleomorphic nuclei and blast-like cells may be present, while some cases may appear like Hodgkin lymphoma with expansion of paracortical areas by small- to medium-sized lymphocytes [108]. On immunohistochemistry, ATL cells express T-cell markers including CD2, CD3, CD4, and CD5, while they are mostly negative for CD8 [108]. 

The current treatment options of ATL include observation, zidovudine and interferon-alfa, chemotherapy, or allogeneic haematopoietic stem cell transplantation [134]. In a meta-analysis of 1767 ATL patients that were managed with allogeneic haematopoietic cell transplantation [135], there was a pooled overall survival of 40% although relapse still occurred in more than one-third of cases. Emerging therapeutic options include the use of anti-metabolites such as cladribine, clofarabine, monoclonal antibodies such as mogamulizumab, proteasome inhibitors, immunomodulators such as lenalidomide, and therapeutic vaccines [134]. 

### 3.6. Merkel Cell Polyomavirus (MCPyV)

MCPyV belongs to the family of human polyomaviruses, along with BKPyV. It is a recently identified virus implicated in the pathogenesis of merkel cell carcinoma (MCC), where the genomic sequences of MCPyV were detected in 80.0% of MCC [12]. It is a double-stranded DNA virus, with an early coding region that expresses three T antigens including large T antigen (LT) and small T antigen (ST) [136,137,138]. Although MCPyV can be found in the skin of the healthy population, most do not develop MCC [137]. The prevalence of MCPyV in MCC is variable depending on the region of interest, with 88.5% of MCC in Japanese patients [139], up to 76.0% in the United States population [140], and 66.6% in Swiss patients [141] found to be positive for MCPyV DNA. In a study of 37 MCC in patients from North America and Australia [142], it was reported that 69.0% of North American patients with MCC were positive for MCPyV DNA, compared to only 24.0% of the Australian patients. The authors postulated that increased sun exposure in Australia may have made viral aetiology a less frequent contributing factor [142]. Aside from MCPyV, ultraviolet exposure and immune deficiencies are also risk factors for the development of MCC [137], with higher prevalence of MCPyV reported in individuals with HIV [143]. A meta-analysis of 22 studies reported a pooled risk ratio of 6.32 for MCC associated with MCPyV although there was also a non-negligible proportion of controls with MCPyV [144]. Clinically, MCC may present like other skin neoplasms, as a rapidly growing skin nodule in sun-damaged skin or may also present as metastatic disease [137].

The pathogenesis of MCC were previously elaborated in greater details [136,137,145,146]. Becker et al. [137] outlined the key pathogenic mechanisms, which are initiated either by “the clonal integration of the MCPyV genome or ultraviolet (UV)-mediated DNA damage caused by chronic exposure to sunlight”. Key mechanisms involved in the development of MCC include the expression of LT and ST proteins, RB1 and TP53 pathways, and UV-induced DNA mutations, although these mechanisms differ depending on the presence of MCPyV [137]. 

Histologically, the tumour is located in the dermis and/or subcutis, with cell size ranging from small to large, and the nuclei showing the characteristic salt-and-pepper chromatin seen in neuroendocrine neoplasms as well as nuclear moulding (Figure 4G,H) [147]. Brisk mitotic activity is often present. On immunohistochemistry, the cells are positive for epithelial markers including CAM 5.2, AE1/3, and CK20, as well as neuroendocrine markers including synaptophysin [137]. A negative TTF-1 differentiates it from a metastatic small cell carcinoma [148]. The MCPyV T antigens can also be highlighted on immunohistochemistry [137]. 

The management of MCC includes wide local excision, assessment of regional lymph nodes with a consideration for sentinel lymph node biopsy if clinically indicated, radiotherapy, chemotherapy, and immunotherapy, with promising results reported in clinical trials investigating the efficacy of anti-PD-L1 therapies such as pembrolizumab [137]. Therapeutic vaccines remain a field that is worth exploring in MCC associated with MCPyV [149].

### 3.7. Helicobacter Pylori (HP)

Helicobacter pylori (H. Pylori) is a member of the Helicobacteraceae family of bacteria. It colonises the gastric epithelium of about half the global population [150]. The bacterium contains urease, which allows it to survive the harsh low pH environment of the stomach and flagella enabling it to eventually migrate and reside in the neutral-pH mucous layer of the epithelium [19]. Of those colonised, approximately 10–15% develop gastritis and peptic ulcer disease, an aetiological link which merited the Nobel Prize in 2005. 

While H. Pylori was first discovered through histologic evaluation of biopsies and microbiologic cultures, many non-invasive diagnostic tools are available today, for instance, urea breath test and stool antigen test [151]. Nevertheless, histologic evaluation remains relevant in patients with indications for endoscopic evaluation (for example, iron deficiency anaemia and alarm symptoms) [151] and can further provide information about degree of inflammation, glandular atrophy and intestinal metaplasia, while facilitating less common but critical diagnoses such as dysplasia, carcinoma, and lymphoma. 

On tissue sections, H. pylori are represented as spiral-shaped organisms, 5µm or less in length, usually located in the lumen of the superficial antral glands. Additional vigilance is required in the detection of these organisms in patients on proton pump inhibitor therapy which allows the organisms to colonise the oxyntic mucosa and the deeper glands. When present in low quantities, immunohistochemistry for H. Pylori may be used as an ancillary diagnostic tool (Figure 4I). Accurate detection of H. Pylori allows the clinician to institute eradication therapy, which typically includes a combination of antibiotics, proton-pump inhibitor and/or antacid (triple or quadruple therapy).

Chronic H. Pylori infection has been implicated in gastric adenocarcinoma (Figure 4J,K), gastric mucosa-associated lymphoid tissue (MALT) lymphoma and diffuse large B-cell lymphoma (DLBCL) (Figure 4L). Gastric carcinogenesis is a complex process contributed by the process of inflammation and epithelial proliferation (similar to that induced by hepatotropic viruses in the liver) as well as H. Pylori strain-specific virulence factors such as cytotoxin associated gene A (CagA) and vacuolating cytotoxin A (VacA) [152]. Successful eradication of H. Pylori in some populations has led to a reduction in rates of gastric cancer, ushering a new era of H. Pylori-negative gastric cancers [153]. Histologically, gastric adenocarcinoma has several subtypes including tubular, papillary, poorly cohesive (signet-ring), mucinous, and mixed subtypes [88], and each subtype shows a characteristic morphologic appearance (Figure 4J,K).

The management of gastric adenocarcinoma includes surgical resection, chemoradiotherapy, targeted therapy, and immunotherapy with the use of agents like pembrolizumab, trastuzumab, and ramucirumab [154]. PD-L1 has been postulated to be involved in the chronicity of H. pylori infection and also in the impaired immune responses against neoplastic cells [155]. Furthermore, H. pylori seropositivity has been reported to be associated with a poorer prognosis in non-small-cell lung cancer patients on PD-L1 blockade therapy [156]. These findings require validation with larger prospective studies. 

### 3.8. Opisthorcis Viverrine (Ov) and Clonorchis Sinensis (Cs)

Ov and Cs are liver flukes, members of the Opisthorchiidae family [157]. Os is prevalent in South East Asia [158], including countries like Thailand, Laos, and Cambodia [159], while Cs is prevalent in East Asia, affecting mainly China, Korea, East Russia, Taiwan, and Vietnam [157]. The tradition of consuming raw fish has accounted for most liver fluke infections in Thailand [158]. Humans are host to Ov, among other hosts, including fresh water intermediate hosts and domesticated cats and dogs [160]. In the infected host, the Ov metacercaria excyst in the duodenum, and ascend into the biliary ducts where they develop to adult worms which then reside in the biliary system of the host [158]. Cs requires three different hosts for its life cycle (snails, fish, and mammals), with similar pattern of migration to biliary epithelium as Ov [157]. 

Ov and Cs infections are typically asymptomatic, but mild symptoms such as abdominal pain and diarrhoea may occur, and more severe symptoms with hepatomegaly and malnutrition may be seen with chronic infections [158,161]. Parasitic infection can be confirmed on stool examination [161] and serological methods using ELISA and DNA-based methods via PCR have also been utilised in the diagnosis of these infections [161]. Eggs of Ov and Cs can be identified microscopically in stool specimens, and they are “operculated and possess prominent opercular ‘shoulders’ and abopercular knob” [162]. The oncogenesis of Ov and Cs can be attributed to three main mechanisms, including mechanical damage to the biliary epithelium, pathological damage induced by inflammation, and the toxic effects of parasite secretions [158,160,163]. The parasitic metabolic products are immunogenic and may interact with biliary epithelium to incite inflammation and promote cellular proliferation [163]. Microarray analysis has revealed upregulation of expression of 131 genes during the development of cholangiocarcinoma induced by Ov infection, and these genes were related to cell proliferation, differentiation, and transformation [164]. Oncogenic pathways to cholangiocarcinoma have been reviewed previously [160,165]. 

**Figure 5:**
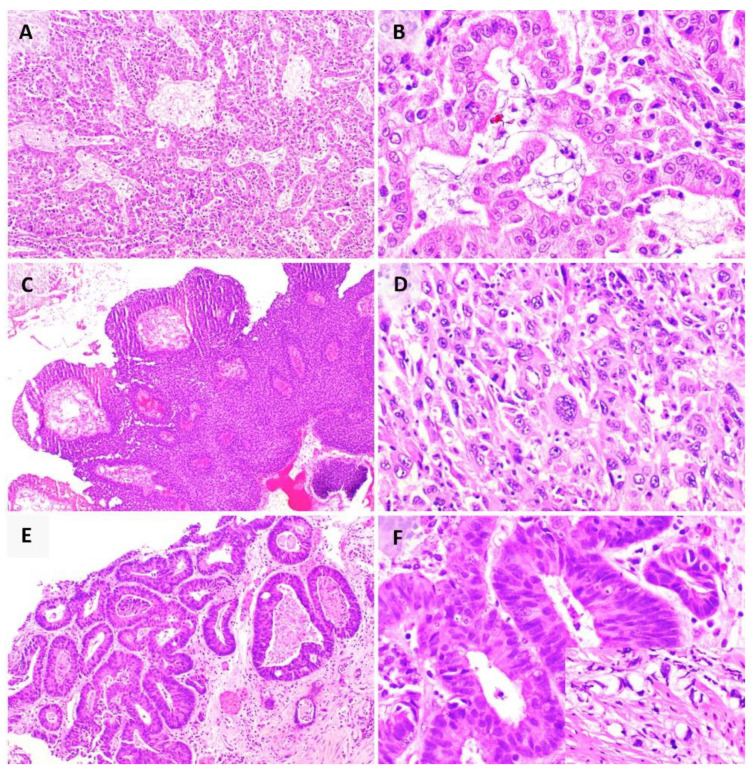
(**A**) Low power magnification of cholangiocarcinoma, with cribriform glands; (**B**) high power magnification of cholangiocarcinoma, with glands lined by cells with marked atypia; (**C**) low power magnification of urothelial carcinoma showing an orderly architecture; (**D**) high power magnification of urothelial carcinoma, with cells showing high grade nuclear features; (**E**) low power magnification of bladder adenocarcinoma, made up of cribriform glands with necrosis present; (**F**) high power magnification of bladder adenocarcinoma, with glands lined by pseudostratified columnar epithelium with hyperchromatic nuclei (inset: signet ring cells, with intracellular vacuolation, can also be seen in adenocarcinoma).

Chronic Ov infection shows characteristic histological changes such as periportal and periductal fibrosis in the liver, and adenomatous hyperplasia and epithelial hyperplasia in the gallbladder [158], while Cs infection shows changes including adenomatous hyperplasia, mucinous metaplasia, periductal inflammation and fibrosis, and dysplasia or neoplasia of biliary cells [157]. Both Ov and Cs infections are associated with the development of extrahepatic and intrahepatic cholangiocarcinoma (cancer of the bile ducts). On histology, (Figure 5A,B) intrahepatic cholangiocarcinomas show a glandular proliferation of small- to medium-sized cuboidal or columnar cells within a desmoplastic stroma [88]. Intrahepatic cholangiocarcinomas can be subdivided into small duct and large duct types, with each having a predilection for either a central or peripheral location and differing based on the morphology and immunohistochemistry of the cells. Extrahepatic cholangiocarcinomas show irregular glands and small clusters of cells [88]. Cholangiocarcinomas are typically positive for cytokeratins CK7, CK19 and negative for CK20 by immunohistochemistry.

The management of cholangiocarcinoma typically involves surgical resection, but most patients are not surgical candidates at the time of presentation and there is limited benefit of systemic chemotherapy [166]. There are emerging therapies aimed at molecular targets including IL-6 [166] and NF-κB [167]. Promising results have been reported in therapy aimed at the fibroblast growth factors pathways [168,169] as well as targets like isocitrate dehydrogenase-1 mutations [169].

### 3.9. Schistosoma Haematobium (Sh)

Sh belongs to the Schistosomatidae family, and are blood flukes [170], located predominantly in the perivesical venous plexus and is implicated in urinary schistosomiasis (bilharziasis) [171]. It is endemic in Africa and the Middle East [171]. These parasites have two hosts, namely, a mammalian host and freshwater snails [170], and humans are infected through skin contact with Sh in freshwater, which then enters the cutaneous venules and lymphatic vessels, ultimately making its way to the peri-vesical venous plexus and veins; many parasitic eggs are trapped in bladder wall and incite immunologic reactions [170,171], such as granulomatous inflammation which can progress to fibrosis and malignancy [172]. Sh infection may be minimally symptomatic [171] or cause clinical manifestations including abdominal pain, diarrhoea, portal hypertension, and cognitive impairment [170]. The pathogenesis and oncogenesis of Sh involves the Sh cell total antigen promoting proliferation of urothelial cells [170,173]. Sh was also postulated to be implicated in the oncogenic mutation of KRAS gene, which is frequently found in bladder carcinomas [174].

The pathological findings of urinary schistosomiasis are related to the inflammatory reactions to parasitic egg deposition [170], and these include fibrosis due to healing granulomata and calcifications. The parasitic eggs can be identified microscopically in stool or urine, and they show a “prominent lateral spine near the posterior end” and a “tapered and slightly curved anterior end” [175]. Sh could also lead to cystitis cystica and cystitis glandularis [170]. Sh infection is associated with bladder carcinoma, primarily urothelial carcinoma (Figure 5C,D), SCC and adenocarcinoma (Figure 5E,F) [170]. As mentioned earlier, SCC is characterised pathologically by a proliferation of squamous cells with intercellular bridges, with or without presence of keratin pearls, denoting the squamous nature of these lesions [176], and adenocarcinomas of the bladder can be subdivided into three subtypes, namely, enteric, mucinous, and mixed types [176]. Adenocarcinomas show immunohistochemical staining for CDX2 and CK20 [176]. 

For treatment of urinary schistosomiasis, praziquantel is the primary therapy but it is ineffective against juvenile schistosomes [177]. Non-invasive bladder carcinoma can be treated with transurethral resection of bladder tumour, adjuvant intravesical chemotherapy and immunotherapy depending on the risk group classification [170]. Radical cystectomy is the mainstay of treatment for invasive bladder carcinomas, with adjuvant or neoadjuvant chemoradiotherapy, depending on stage of cancer [170]. There is emerging evidence that immune checkpoint inhibitors such as pembrolizumab improve prognosis in patients with locally advanced or metastatic urothelial carcinoma with progression after chemotherapy [178]. Vaccine developments have proven to be challenging [177]. 

### 3.10. COVID-19

At the time of writing, there are over 200 million cases of COVID-19 worldwide [179]. Predominantly an infection of the respiratory tract, it manifests, in severe cases, as acute respiratory distress syndrome [180]. The association of a deregulated immune response and ground glass changes on imaging with COVID-19 infection has been postulated to facilitate cancer initiation and progression [181]. COVID-19 and cancer also involve common signalling pathways including cytokine signalling, IL-6 and JAK/STAT signalling, and immune checkpoint signalling amongst numerous other pathways [15]. Several viral infections such as HIV and HBV also show high expression of programmed cell death-1 receptor [15] and there is also emerging evidence that immune checkpoint receptors are upregulated in severe COVID-19 cases [182]. Although this evidence demonstrates similarities between COVID-19 and other pathogens, the true clinical significance of these similarities remains to be determined. With accelerated research and development of novel COVID-19 vaccines [183], it is hoped this knowledge can be used to develop similar vaccines against other familiar pathogens. 

## 4. Conclusions

Pathogens are frequently encountered in the practice of anatomical pathology, with some presenting as benign mimickers of malignancies and others as more commonly seen malignancies. Considering many of the oncogenic pathogens are endemic in certain parts of the world and are often asymptomatic, there may be a greater need for early detection of these pathogens as well as identification of the pre-malignant manifestations to initiate prompt medical intervention. With increasing attention given to personalised medicine, molecular diagnostics and therapeutics, it is likely that more knowledge will be uncovered about the oncogenic molecular pathways associated with the various oncogenic pathogens. Furthermore, with an accelerated growth in the research around anti-viral and anti-cancer vaccines, it is envisioned that more will be known about these pathogens and how they may be eradicated. 

## Data Availability

Not applicable.
